# Neonatal encephalopathy: Case definition & guidelines for data collection, analysis, and presentation of maternal immunisation safety data

**DOI:** 10.1016/j.vaccine.2017.01.045

**Published:** 2017-12-04

**Authors:** Erick Sell, Flor M. Munoz, Aung Soe, Max Wiznitzer, Paul T. Heath, E.D. Clarke, Hans Spiegel, Daphne Sawlwin, Maja Šubelj, Ilia Tikhonov, Khorshid Mohammad, Sonali Kochhar, for The Brighton Collaboration Acute Neonatal Encephalopathy Working Group

**Affiliations:** a Children’s Hospital of Eastern Ontario, Canada; b Baylor College of Medicine, United States; c Medway Maritime Hospital, Gillingham, Kent, United Kingdom; d Rainbow Babies and Children’s Hospital in Cleveland, United States; e St. Georges Vaccine Institute, Institute of Infection & Immunity, St. Georges, University of London, London, UK; f Medical Research Unit, Gambia; g Henry M. Jackson Foundation, Kelly Government Solutions (KGS), Contractor to DAIDS/NIAID/NIH, Rockville, United States; h Therapeutic Area Lead, Vaccines, Safety Risk Management, Australian QPPVCSL Limited Melbourne, Australia; i National Institute of Public Health, Ljubljana, Slovenia; j Sanofi Pasteur, United States; k University of Calgary, Section of Neonatology, Department of Pediatrics, Foothills Medical Centre, Canada; l Global Healthcare Consulting, Delhi, India; m Erasmus University Medical Center, Rotterdam, The Netherlands

**Keywords:** Neonatal encephalopathy, Adverse event, Immunisation, Guidelines, Case definition

## 1 Preamble

### 1.1 Background on neonatal encephalopathy

To improve comparability of vaccine safety data, the acute neonatal encephalopathy working group has developed a case definition and guidelines neonatal encephalopathy applicable in study settings with different availability of resources, in healthcare settings that differ by availability of and access to health care, and in different geographic regions.

The definition and guidelines were developed through group consensus. They are grounded on both expert opinion and a systematic literature review related to the assessment of acute neonatal encephalopathy as an adverse event following immunisation and to the diagnosis of acute neonatal encephalopathy in humans.

Encephalopathy is a general term used to define disease, malfunction or damage of the brain. The major symptom of encephalopathy is an altered mental state [1]. Defining altered mental state in the newborn is significantly more challenging than in the adult and there are no established direct measures to determine level of consciousness in the newborn. Nevertheless, specific clinical signs reflecting neurological function correlate with the overall severity of the encephalopathy. These clinical signs have been grouped in stages, usually three of them: mild, moderate and severe as in the Sarnat classification. The Sarnat criteria remain as the most commonly accepted classification [2], [3].

Neonatal encephalopathy has several potential etiologies and acute hypoxia-ischemia is the most studied cause. Over the years, the term “neonatal encephalopathy”, has been used by many as a synonym of “Hypoxic-ischemic encephalopathy”. The reason is that other etiologies are often reported as a specific diagnosis, as in the case of inborn errors of metabolism (e.g. non-ketotic hyperglycinemia), infections (e.g. meningitis) and other specific causes. It is therefore imperative to emphasize that many different processes leading to neonatal encephalopathy may develop prenatally, at birth or immediately post-delivery, and result from mainly, but not exclusively, genetic, metabolic, infectious, and traumatic processes. The common denominator in neonatal encephalopathy is the loss of homeostasis which can lead to abnormal brain function and potentially to brain structural changes [2], [3], [4].

Investigations such as magnetic resonance Imaging and neurophysiological technologies such as electroencephalography and evoked potentials are aids in exploring the severity and prognosis of neonatal encephalopathy, but are not required for its diagnosis. MRI brain can be reported normal in 15–30% of cases of confirmed mild cases (Sarnat 1) of hypoxic ischemic encephalopathy [1], [5].

The EEG is the most specific test to confirm and diagnose that a clinical paroxysm is epileptic in origin [9]. Electrographic pathological patterns correlate with neonatal seizures during seizure recording and subtle or subclinical seizures can often only be diagnosed by EEG monitoring. Once anti-seizure medications are administered, up to 58% of treated neonates exhibit electro-clinical uncoupling, in which the clinical signs of their seizures vanish despite the persistence of subclinical electrographic seizures [9].

EEG however has important technical limitations in the recording of some epileptic seizures, particularly those originating in mesial or midline areas of the brain. Also EEG is not always readily available for recording in the NICU. Amplitude integrated EEG (aEEG) is becoming widely used by neonatologists. This recording compresses the time scale of conventional EEG. It has lesser spatial resolution and is less sensitive for the detection of neonatal seizures compared to long term monitoring by conventional EEG [10], [11]. Abnormalities on the neonatal EEG such as discontinuity of the background or central sharp waves are not specific and will vary depending on external factors such as gestational age, body temperature during therapeutic cooling and medications [7].

Therefore, as per World Health Organization guidelines on neonatal seizures, the most practical method of diagnosis is the clinical recognition of neonatal seizures [8].

The Task Force on Neonatal Encephalopathy group on Neonatal Encephalopathy and Neurological outcome published in 2014 a comprehensive document defining Neonatal encephalopathy as “a clinical syndrome in an infant born at or beyond 35 weeks of gestation, manifested by a subnormal level of consciousness or seizures, and often accompanied by difficulty with initiating and maintaining respiration and depression of tone and reflexes” [5]. It was the consensus of our group to replace the word “consciousness” with the word “alertness” since the definition of “consciousness” is more vague. We also considered it necessary to include in the definition the concept of multiple potential etiologies.

When assessing encephalopathy in a newborn younger than 35 weeks of gestation, normal neurological development may prevent specific behavioral reactions and reflexes to be tested, making semiology unreliable. Furthermore, the diagnosis of “encephalopathy of prematurity” is heavily based on neuroanatomical changes seen on MRI or autopsies rather than acute clinical signs [2], [6].

Considering the limitations for diagnosing and timing encephalopathies in the preterm newborn, this review defines Neonatal encephalopathy as a clinical syndrome presenting with abnormal functioning of the central nervous system, in the earliest days of life in an newborn (up to 28 days of life) born at or beyond 35 weeks of gestation, manifested by an abnormal level of alertness or seizures, and often accompanied by difficulty with initiating and maintaining respiration and depression of tone that may be due to a variety of etiologies including hypoxia/ischemia, metabolic disturbance, or infection.

This definition is to be equally applied in vaccinated or unvaccinated populations.

### 1.2 Methods for the development of the case definition and guidelines for data collection, analysis, and presentation for neonatal encephalopathy as an adverse events following maternal immunisation

Following the process described on the Brighton Collaboration Website http://www.brightoncollaboration.org/internet/en/index/process.html, the Brighton Collaboration Neonatal Encephalopathy *Working Group* was formed in 2016 and included members of clinical, academic, public health and industry background. The composition of the working and reference group as well as results of the web-based survey completed by the reference group with subsequent discussions in the working group can be viewed at: http://www.brightoncollaboration.org/internet/en/index/working_groups.html.

To guide the decision-making for the case definition and guidelines, a literature search was performed using Embase.com (Medline/PubMed + Embase); ClinicalKey (eBooks); ScienceDirect (eBooks); StatRef (eBooks) and the Cochrane Library.

Several different research platforms were utilized in this search for references focused on maternal vaccination and encephalopathies. These platforms included electronic books, systematic reviews, and other journal literature. The following three search parameters which included a variety of synonyms were combined; pregnancy, vaccines, and encephalopathy.

This search resulted in several general book chapters discussing various types of neonatal encephalopathy. There were no Cochrane reviews that focused on this topic. The journal literature search produced 33 results that included subject headings or keywords for vaccines, pregnancy and encephalopathy. The results were limited to those published since 2005. The results were further limited to either reviews or major/prospective clinical studies.

### 1.3 Temporal versus causal association with maternal immunisation

There are no reports of encephalopathy following immunisation in the pregnant woman or the newborn. There is hence no uniformly accepted definition of Neonatal Encephalopathy following immunisations. This is a missed opportunity, as data comparability across trials or surveillance systems would facilitate data interpretation and promote the scientific understanding of the event.

### 1.4 Periodic review

Similar to all Brighton Collaboration case definitions and guidelines, review of the definition with its guidelines is planned on a regular basis (i.e. every three to five years) or more often if needed.

## 2 Case definition of neonatal encephalopathy^3^

**For All Levels of Diagnostic Certainty**

Newborn (1–28 days) born at or beyond 35 weeks of gestation

**Level 1 of diagnostic certainty (Definite)**

Abnormal level of alertness or seizures (see footnote 1)

**AND**

Difficulty with initiating and maintaining respiration

**AND**

Depression of tone

**Level 2 of diagnostic certainty (Probable)**

Abnormal level of alertness or seizures

**AND**

Difficulty with initiating and maintaining respiration

**OR**

Depression of tone

**Level 3 of diagnostic certainty (Possible)**

Abnormal level of alertness or seizures without difficulty with initiating and maintaining respiration nor depression of tone

## 3 Guidelines for data collection, analysis, and presentation of generalized acute neonatal encephalopathy as an adverse event following maternal immunisation

It was the consensus of the Brighton Collaboration Neonatal Encephalopathy *Working Group* to recommend the following guidelines to enable meaningful and standardized collection, analysis, and presentation of information about Neonatal encephalopathy. However, implementation of all guidelines might not be possible in all settings. The availability of information may vary depending upon resources, geographical region, and whether the source of information is a prospective clinical trial, a post-marketing surveillance or epidemiological study, or an individual report of Neonatal encephalopathy. Also, as explained in more detail in the overview paper, these guidelines have been developed by this working group for guidance only, and are not to be considered a mandatory requirement for data collection, analysis, or presentation.

### 3.1 Data collection

These guidelines represent a desirable standard for the collection of data on availability following maternal immunisation to allow for comparability of data, and are recommended as an addition to data collected for the specific study question and setting. The guidelines are not intended to guide the primary reporting of Neonatal Encephalopathy to a surveillance system or study monitor. Investigators developing a data collection tool based on these data collection guidelines also need to refer to the criteria in the case definition, which are not repeated in these guidelines.

#### 3.1.1 Source of information/reporter

For all cases and/or all study participants, as appropriate, the following information should be recorded:

(1) Date of report.
(2) Name and contact information of person reporting^4^ and/or diagnosing the Neonatal Encephalopathy as specified by country-specific data protection law.
(3) Name and contact information of the investigator responsible for the subject, as applicable.
(4) Relation to the patient (e.g., immuniser [clinician, nurse], family member [indicate relationship], other).

#### 3.1.2 Vaccinee/control

##### 3.1.2.1 Demographics

For all cases and/or all study participants (including mothers and infants), as appropriate, the following information should be recorded:

(5) Case/study participant identifiers (e.g. first name initial followed by last name initial) or code (or in accordance with country-specific data protection laws).
(6) Date of birth, age, and sex.
(7) For infants: Gestational age and birth weight.

##### 3.1.2.2 Clinical and immunisation history

For all cases and/or all study participants, as appropriate, the following information should be recorded:

(8) Past medical history, including hospitalisations, underlying diseases/disorders, pre-immunisation signs and symptoms including identification of indicators for, or the absence of, a history of allergy to vaccines, vaccine components or medications; food allergy; allergic rhinitis; eczema; asthma.
(9) Any medication history (other than treatment for the event described) prior to, during, and after immunisation including prescription and non-prescription medication as well as medication or treatment with long half-life or long term effect. (e.g. immunoglobulins, blood transfusion and immunosuppressants).
(10) Immunisation history (i.e. previous immunisations and any adverse event following immunisation (AEFI)), in particular occurrence of Acute Neonatal Encephalopathy after a previous maternal immunisation.

#### 3.1.3 Details of the immunisation

For all cases and/or all study participants, as appropriate, the following information should be recorded:

(11) Date and time of maternal immunisation(s).
(12) Description of vaccine(s) (name of vaccine, manufacturer, lot number, dose (e.g. 0.25 mL, 0.5 mL, etc.) and number of dose if part of a series of immunisations against the same disease).
(13) The anatomical sites (including left or right side) of all immunisations (e.g. vaccine A in proximal left lateral thigh, vaccine B in left deltoid).
(14) Route and method of administration (e.g. intramuscular, intradermal, subcutaneous, and needle-free (including type and size), other injection devices).
(15) Needle length and gauge.

#### 3.1.4 The adverse event

(16) For all cases at any level of diagnostic certainty and for reported events with insufficient evidence, the criteria fulfilled to meet the case definition should be recorded.

Specifically document:

(17) Clinical description of signs and symptoms of Neonatal Encephalopathy, and if there was medical confirmation of the event (i.e. patient seen by physician).
(18) Date/time of onset,^5^ first observation^6^ and diagnosis,^7^ end of episode^8^ and final outcome.^9^
(19) Concurrent signs, symptoms, and diseases.
(20) Measurement/testing
  • Values and units of routinely measured parameters (e.g. temperature, blood pressure) – in particular those indicating the severity of the event;
  • Method of measurement (e.g. type of thermometer, oral or other route, duration of measurement, etc.);
  • Results of laboratory examinations, surgical and/or pathological findings and diagnoses if present.
(21) Treatment given for Neonatal Encephalopathy, especially specify what and dosing.
(22) Outcome (see footnote 8) at last observation.
(23) Objective clinical evidence supporting classification of the event as “serious”.^10^
(24) Exposures other than the immunisation 24 h before and after immunisation (e.g. food, environmental) considered potentially relevant to the reported event.

#### 3.1.5 Miscellaneous/general

(25) The duration of surveillance for Neonatal encephalopathy should be predefined based on
  • Biologic characteristics of the vaccine e.g. live attenuated versus inactivated component vaccines;
  • Biologic characteristics of the vaccine-targeted disease;
  • Biologic characteristics of Neonatal encephalopathy including patterns identified in previous trials (e.g. early-phase trials); and
  • Biologic characteristics of the vaccinee (e.g. nutrition, underlying disease like immunodepressing illness).
(26) The duration of follow-up reported during the surveillance period should be predefined likewise. It should aim to continue to resolution of the event.
(27) Methods of data collection should be consistent within and between study groups, if applicable.
(28) Follow-up of cases should attempt to verify and complete the information collected as outlined in data collection guidelines 1–24.
(29) Investigators of patients with Neonatal encephalopathy should provide guidance to reporters to optimise the quality and completeness of information provided.
(30) Reports of Neonatal encephalopathy should be collected throughout the study period regardless of the time elapsed between immunisation and the adverse event. If this is not feasible due to the study design, the study periods during which safety data are being collected should be clearly defined.

### 3.2 Data analysis

The following guidelines represent a desirable standard for analysis of data on Neonatal encephalopathy to allow for comparability of data, and are recommended as an addition to data analysed for the specific study question and setting.

(31) Reported events should be classified in one of the following five categories including the three levels of diagnostic certainty. Events that meet the case definition should be classified according to the levels of diagnostic certainty as specified in the case definition. Events that do not meet the case definition should be classified in the additional categories for analysis.

#### 3.2.1 Event classification in 5 categories^11^

##### 3.2.1.1 Event meets case definition

(1) Level 1: Criteria as specified in the Neonatal encephalopathy case definition (Definite)
(2) Level 2: Criteria as specified in the Neonatal encephalopathy case definition (Possible)
(3) Level 3: Criteria as specified in the Neonatal encephalopathy case definition (Probable)

#### 3.2.2 Event does not meet case definition

##### 3.2.2.1 Additional categories for analysis

(4) Reported Neonatal encephalopathy with insufficient evidence to meet the case definition^12^
(5) Not a case of Neonatal encephalopathy^13^

(32) The interval between immunisation and reported Neonatal Encephalopathy could be defined as the date/time of maternal immunisation to the date/time of onset (see footnote 4) of the first symptoms and/or signs consistent with the definition.
(33) The duration of a possible Neonatal Encephalopathy could be analysed as the interval between the date/time of onset (see footnote 3) of the first symptoms and/or signs consistent with the definition and the end of episode (see footnote 7) and/or final outcome (see footnote 8). Whatever start and ending are used, they should be used consistently within and across study groups.
(34) If more than one measurement of a particular criterion is taken and recorded, the value corresponding to the greatest magnitude of the adverse experience could be used as the basis for analysis. Analysis may also include other characteristics like qualitative patterns of criteria defining the event.
(35) The distribution of data (as numerator and denominator data) could be analysed in predefined increments (e.g. measured values, times), where applicable. Increments specified above should be used. When only a small number of cases is presented, the respective values or time course can be presented individually.
(36) Data on Neonatal Encephalopathy obtained from subjects receiving a vaccine should be compared with those obtained from an appropriately selected and documented control group(s) to assess background rates of hypersensitivity in non-exposed populations, and should be analysed by study arm and dose where possible, e.g. in prospective clinical trials.

### 3.3 Data presentation

These guidelines represent a desirable standard for the presentation and publication of data on Neonatal Encephalopathy following maternal immunisation to allow for comparability of data, and are recommended as an addition to data presented for the specific study question and setting. Additionally, it is recommended to refer to existing general guidelines for the presentation and publication of randomised controlled trials, systematic reviews, and meta-analyses of observational studies in epidemiology (e.g. statements of Consolidated Standards of Reporting Trials (CONSORT), of improving the quality of reports of meta-analyses of randomised controlled trials (QUORUM), and of meta-analysis Of Observational Studies in Epidemiology (MOOSE), respectively).

(37) All reported events of Neonatal Encephalopathy should be presented according to the categories listed in guideline 31.
(38) Data on Neonatal Encephalopathy events should be presented in accordance with data collection guidelines 1–24 and data analysis guidelines 31–36
(39) Terms to describe Neonatal Encephalopathy such as “low-grade”, “mild”, “moderate”, “high”, “severe” or “significant” are highly subjective, prone to wide interpretation, and should be avoided, unless clearly defined.
(40) Data should be presented with numerator and denominator (n/N) (and not only in percentages), if available.

Although immunisation safety surveillance systems denominator data are usually not readily available, attempts should be made to identify approximate denominators. The source of the denominator data should be reported and calculations of estimates be described (e.g. manufacturer data like total doses distributed, reporting through Ministry of Health, coverage/population based data, etc.).

(41) The incidence of cases in the study population should be presented and clearly identified as such in the text.
(42) If the distribution of data is skewed, median and range are usually the more appropriate statistical descriptors than a mean. However, the mean and standard deviation should also be provided.
(43) Any publication of data on Neonatal encephalopathy should include a detailed description of the methods used for data collection and analysis as possible. It is essential to specify:
• The study design;
• The method, frequency and duration of monitoring for Acute Neonatal encephalopathy;
• The trial profile, indicating participant flow during a study including drop-outs and withdrawals to indicate the size and nature of the respective groups under investigation;
• The type of surveillance (e.g. passive or active surveillance);
• The characteristics of the surveillance system (e.g. population served, mode of report solicitation);
• The search strategy in surveillance databases;
• Comparison group(s), if used for analysis;
• The instrument of data collection (e.g. standardised questionnaire, diary card, report form);
• Whether the day of immunisation was considered “day one” or “day zero” in the analysis;
• Whether the date of onset (see footnote 4) and/or the date of first observation (see footnote 5) and/or the date of diagnosis (see footnote 6) was used for analysis; and
• Use of this case definition for Neonatal encephalopathy, in the abstract or methods section of a publication.^14^

## Disclaimer

The findings, opinions and assertions contained in this consensus document are those of the individual scientific professional members of the working group. They do not necessarily represent the official positions of each participant’s organisation (e.g., government, university, or corporation). Specifically, the findings and conclusions in this paper are those of the authors and do not necessarily represent the views of their respective institutions.

## Footnotes

3 For Seizure definition see Ref. [12].

4 If the reporting centre is different from the vaccinating centre, appropriate and timely communication of the adverse event should occur.

5 The date and/or time of onset is defined as the time post immunisation, when the first sign or symptom indicative for Neonatal Encephalopathy occurred. This may only be possible to determine in retrospect.

6 The date and/or time of first observation of the first sign or symptom indicative for Neonatal Encephalopathy can be used if date/time of onset is not known.

7 The date of diagnosis of an episode is the day post immunisation when the event met the case definition at any level.

8 The end of an episode is defined as the time the event no longer meets the case definition at the lowest level of the definition.

9 E.g. recovery to pre-immunisation health status, spontaneous resolution, therapeutic intervention, persistence of the event, sequelae, death.

10 An AEFI is defined as serious by international standards if it meets one or more of the following criteria: (1) it results in death, (2) is life-threatening, (3) it requires inpatient hospitalisation or results in prolongation of existing hospitalisation, (4) results in persistent or significant disability/incapacity, (5) is a congenital anomaly/birth defect, and (6) is a medically important event or reaction.

11 To determine the appropriate category, the user should first establish, whether a reported event meets the criteria for the lowest applicable level of diagnostic certainty, e.g. Level three. If the lowest applicable level of diagnostic certainty of the definition is met, and there is evidence that the criteria of the next higher level of diagnostic certainty are met, the event should be classified in the next category. This approach should be continued until the highest level of diagnostic certainty for a given event could be determined.

12 If the evidence available for an event is insufficient because information is missing, such an event should be categorised as “Reported Neonatal Encephalopathy with insufficient evidence to meet the case definition”.

13 An event does not meet the case definition if investigation reveals a negative finding of a necessary criterion (necessary condition) for diagnosis. Such an event should be rejected and classified as “Not a case of Neonatal Encephalopathy”.

14 Use of this document should preferably be referenced by referring to the respective link on the Brighton Collaboration website (http://www.brightoncollaboration.org).
